# Mesenchymal stem cells from *Cerdocyon thous*: in vitro characterization and therapeutic potential

**DOI:** 10.1007/s11259-026-11542-4

**Published:** 2026-09-26

**Authors:** João Vinícius Carneiro de Aragão, Joshua Benjamín Andrés Polanco Stuart, Thaís Gonçalves Tavares, Lucas Vinícius de Oliveira Ferreira, Aline Márcia Marques Braz, Marjorie de Assis Golim, Márcio de Carvalho, Rogério Martins Amorim

**Affiliations:** 1 Department of Veterinary Clinic, School of Veterinary Medicine and Animal Science, São Paulo State University (UNESP), Botucatu, SP 18618-681 Brazil; 2 Center for Translational Research in Regenerative Medicine, Institute of Biotechnology, São Paulo State University (UNESP), Botucatu, SP 18607-440 Brazil; 3 Laboratory of Applied Biotechnology, Clinical Hospital of the Medical School, São Paulo State University (UNESP), Botucatu, SP 18618-687 Brazil; 4 Center for the Study of Venoms and Venomous Animals (CEVAP), São Paulo State University (UNESP), Botucatu, SP 18619002 Brazil

**Keywords:** Immunomodulation, Neuroregeneration, Conservation medicine, Wild animals

## Abstract

Mesenchymal stem cells (MSCs) exhibit immunomodulatory and regenerative properties that have been extensively investigated in domestic species. However, knowledge of their biological characteristics in wildlife species remains limited and is critical for advancing translational research and conservation medicine. Thus, this study aimed to isolate, characterize, and evaluate the immunomodulatory and neuroregenerative potential of adipose tissue-derived MSCs (AT-MSCs) from crab-eating foxes (*Cerdocyon thous*). Subcutaneous adipose tissue samples were collected from five healthy adult animals during elective surgeries, following prior ethical approval. All procedures were conducted in accordance with institutional and national ethical guidelines. MSCs were isolated by enzymatic digestion, expanded in vitro, and characterized based on morphology, immunophenotype, and mesodermal multipotency. Cell viability was assessed following inflammatory stimulation with lipopolysaccharide (LPS), and the gene expression of immunomodulatory cytokines and neurotrophic factors was evaluated by RT-qPCR. The cells displayed typical fibroblast-like morphology, plastic adherence, expression of mesenchymal markers CD90, CD29, and CD44, absence of hematopoietic markers CD14 and CD45, and successful differentiation into adipogenic, chondrogenic, and osteogenic lineages. LPS stimulation significantly upregulated the expression of indoleamine-2,3-dioxygenase (IDO), as well as the neurotrophic and regenerative factors glial cell line-derived neurotrophic factor (GDNF), hepatocyte growth factor (HGF), and vascular endothelial growth factor A (VEGF-A). These findings indicate that AT-MSCs derived from *Cerdocyon thous* possess immunomodulatory and neuroregenerative potential, supporting their relevance for translational research and future applications in conservation veterinary medicine, particularly in the context of inflammatory and neurological disorders.

**Supplementary Information:**

The online version contains supplementary material available at https://doi.org/10.1007/s11259-026-11542-4.

## Introduction

Characterized by their self-renewal potential, specific immunophenotype, and fibroblastoid morphology, mesenchymal stem cells (MSCs) consist of adherent multipotent cells derived from tissues of mesenchymal origin, with the capacity for adipogenic, chondrogenic, and osteogenic differentiation. Additionally, studies in different species have demonstrated that MSCs also exhibit the potential for transdifferentiation into neural cells such as neural-like cells and Schwann-like cells (Echeverry et al. 2019; Villagrán et al. 2016; Wang et al. 2022).

MSCs can be isolated from various adult tissues, including bone marrow (Friedenstein et al. 1970), skin (Toma et al. 2001), liver (Götherström et al. 2003), synovium (Djouad et al. 2005), dental pulp (Perry et al. 2008), muscle (Kisiel et al. 2012), adipose tissue (Echeverry et al. 2020) and endometrium (Wang et al. 2022), as well as from perinatal tissues such as the umbilical cord and Wharton’s jelly (Ryu et al. 2012), fetal membranes (Andrzejewska et al. 2019) and placenta (Ha et al. 2026). Among these sources, adipose tissue stands out as one of the most promising, particularly in studies involving wild animals, as it allows the acquisition of large quantities of cells through minimally invasive methods or opportunistic collection during elective surgical procedures such as castration (Echeverry et al. 2019).

The therapeutic potential of MSCs is associated with multiple mechanisms of action, including the ability to migrate to injured tissues, differentiate, and interact with the local microenvironment, thereby contributing to tissue homeostasis (Liu et al. 2023). In addition, MSCs exert immunomodulatory effects via interactions with components of the immune system and the secretion of extracellular vesicles and soluble mediators, including cytokines with pro- or anti-inflammatory functions depending on the microenvironmental context (Alvites et al. 2022; Johnson 2024). Their regenerative potential is further supported by the paracrine release of neurotrophic factors such as brain-derived neurotrophic factor (BDNF), nerve growth factor (NGF), and neurotrophin-3 (NT-3) (Colbath et al. 2024). In addition, autocrine signaling contributes to MSC regenerative functions by regulating cellular processes such as migration, survival and differentiation (Hemmingsen et al. 2013; Hoseini and Montazeri 2025; Pelagalli et al. 2018).

Due to these properties, MSCs have been applied in the treatment of various diseases in domestic species, including cardiovascular, respiratory, immune-mediated and renal disorders (Johnson 2024). Similarly, they have been widely investigated as a therapeutic alternative for diseases affecting the nervous system. In equines, the use of MSCs has been explored for conditions of an inflammatory or degenerative nature, with a focus on immunomodulation, neuroprotection, and improvement of the neural microenvironment (Guest et al. 2008; Carrade et al. 2012; Dudhia and Guest 2014). In other domestic animals, their use has been reported in peripheral neuropathies, spinal cord injuries, intervertebral disc disease, meningoencephalomyelitis of unknown origin (MUO), encephalitis caused by the canine distemper virus, cognitive dysfunction, among others (Gonçalves et al. 2018; Pinheiro et al. 2019; Brunel et al. 2022; Altamirano-Samaniego et al. 2022; Johnson 2024; Amorim and Ferreira 2025).

The crab-eating fox (*Cerdocyon thous*) is a Neotropical omnivorous mesocarnivore with predominantly crepuscular and nocturnal habits, and a wide distribution across South America. The species is considered a generalist and highly flexible in its use of habitat (Santos et al. 2024). Processes such as deforestation, agricultural expansion, habitat fragmentation, extinction of top predators, and chronic anthropogenic disturbances (increased human population density, grazing, wildfires, and road collisions) have altered the population dynamics of the species, favouring its approach to anthropogenic environments (Santos et al. 2024). This scenario intensifies the interaction between *C. thous* and domestic animals, especially dogs, increasing the exposure of wild populations to infectious and parasitic agents, with an elevated risk of spillover events, including those involving pathogens with tropism for the central nervous system (Curi et al. 2010).

Among the diseases described in *C. thous*, those involving neurological impairment stand out, particularly canine distemper (CDV). This disease can extensively affect the central nervous system, resulting in diffuse demyelination and a variety of clinical manifestations, such as ataxia, paralysis, plegia, muscle atrophy, hyperesthesia, myoclonus, tremors, and epileptic seizures (Brunel et al. 2022). Fadrique et al. (2025) reported a case of *C. thous* presenting with apathy, dehydration, bilateral purulent ocular discharge, and motor incoordination, in which haematological examinations revealed normocytic normochromic anemia, a degenerative left shift, leukopenia due to lymphopenia, monocytosis, and hypoalbuminemia. The diagnosis of canine distemper was confirmed using an immunochromatographic assay for the detection of viral antigens. Additionally, there are reports of the occurrence of canine distemper in other wild canids, such as the pampas fox (*Lycalopex gymnocercus*) (Hübner et al. 2010), hoary fox (*Lycalopex vetulus*) (Megid et al. 2010), maned wolf (*Chrysocyon brachyurus*) (Furtado et al. 2016), and short-eared dog (*Atelocynus microtis*) (Jucá et al. 2022), reinforcing the relevance of this disease in the context of conservation.

Considering the immunomodulatory, anti-inflammatory, and regenerative potential of MSCs, they represent a promising approach for treating neurological diseases in wild animals. However, although they have been widely characterized in domestic species, such as canines (Rashid 2021), sheep (Dar et al. 2021), swine (Garcia et al. 2022), equines (Arnhold et al. 2007), bovines (Merlo et al. 2022), and felines (Park and Song 2024), there is still a scarcity of information regarding the morphology, biological properties, and functions of MSCs in wild species (Pereira et al. 2018).

Despite the growing interest in regenerative medicine for wildlife species, to the best of our knowledge, there are currently no published studies describing or characterizing MSCs derived from the crab-eating fox (*C. thous*). In this context, the present study aimed to evaluate the anti-inflammatory, immunomodulatory, and neuroregenerative potential of MSCs derived from the adipose tissue (AT-MSCs) of crab-eating foxes (*C. thous*), through their isolation, morphological, immunophenotypic, and multipotential characterization, assessment of cell viability, and analysis of gene expression associated with these processes.

## Materials and methods

### Isolation and culture of mesenchymal stem cells (MSCs)

Excisional collection of abdominal subcutaneous adipose tissue was performed from five *C. thous*, comprising three females and two males, all adults and clinically healthy, maintained under human care at the Center for Medicine and Research in Wild Animals of the School of Veterinary Medicine and Animal Science, Botucatu campus, São Paulo, Brazil, under approval of the Animal Ethics Committee (CEUA) – Protocol: 000.341. The study was conducted from March 2025 to March 2026. Briefly, the animals (*n* = 5) received pre-anesthetic medication consisting of ketamine (10 mg/kg, intramuscular [IM]), midazolam (0.3 mg/kg, IM) and methadone (0.2 mg/kg, IM). The animals were transferred to the surgical center, where anesthesia was induced with propofol (5 mg/kg, intravenous [IV]). Local anesthetic blockade was performed using lidocaine (3 mg/kg, subcutaneously) at the incision site. For postoperative analgesia, the animals received meloxicam (0.1 mg/kg, oral) and dipyrone (25 mg/kg, oral). All procedures were conducted in accordance with the ARRIVE guidelines.

Initially, at the Translational Nucleus of Regenerative Medicine (NUTRAMERE) of the School of Veterinary Medicine and Animal Science, São Paulo State University (UNESP), Botucatu campus, the adipose tissue was fragmented into small pieces in a laminar flow hood and subjected to enzymatic digestion at 37 °C for 2 h using 0.1% Type I collagenase (Thermo Fisher Scientific, Waltham, MA, USA) solution. After digestion, the material was inactivated with DMEM (Dulbecco’s Modified Eagle Medium) supplemented with 10% fetal bovine serum (FBS) and centrifuged to remove the remaining lipid layer. The resulting cell pellet was washed twice with phosphate-buffered saline (PBS) to ensure the complete removal of residual debris. Following washing, the cells were plated in T25 flasks with standard culture medium composed of 90% DMEM, 10% FBS, 1% penicillin–streptomycin, and 1% amphotericin B.

### Morphological, immunophenotypic, and multipotency characterization

For morphological characterization, culture flasks were maintained in an incubator at 37 °C under a 5% CO₂ atmosphere until the cells reached 80–90% confluence over three passages (P3). Cell morphology was documented by optical microscopy using an inverted Nikon Eclipse microscope equipped with a Nikon D5100 digital camera (Nikon Corporation, Tokyo, Japan).

Immunophenotypic characterization of MSCs was performed when P3 cultures reached 80–90% confluence. The surface antigen expression profile was assessed using the markers CD44⁺, CD90⁺, CD29⁺, CD14⁻, and CD45⁻. Cells were incubated with specific monoclonal antibodies: mouse anti-human CD90-APC (BD Pharmingen, New Jersey, USA), mouse anti-human CD14-APC (BD Pharmingen, New Jersey, USA), mouse anti-human CD29-APC (eBioscience, California, USA), rat anti-dog CD45-FITC (eBioscience, California, USA), and rat anti-mouse CD44-PE (BD Pharmingen, New Jersey, USA), according to manufacturer’s protocols. The assessment of antibody cross-reactivity was conducted using peripheral blood from *Cerdocyon thous* (following protocol with red blood cell lysis and subsequent washing). The positive staining with anti-CD45 and anti-CD14 (monocytes) antibodies confirmed the cross-reactivity of these antibodies with the species under study.

Briefly, 100 µL aliquots of the cell suspension (1 × 10⁶ cells/mL in PBS) were transferred to polystyrene tubes. A tube containing only cells was used as an autofluorescent control. The pre-titrated volume of each antibody was added to the remaining tubes. The samples were mixed by vortexing and incubated for 30 min at room temperature, protected from light. After incubation, 500 µL of PBS was added, followed by vortexing and centrifugation at 290 × g for 5 min. The supernatant was discarded, and the cells were resuspended in 500 µL of PBS for data acquisition using a FACSCalibur^®^ flow cytometer (BD Biosciences). The acquisition and analysis of data was conducted using CellQuest™ and FlowJo™ software, employing the following analytical strategy: the MSC cell population was established based on morphological parameters [size (FSC) versus internal complexity (SSC)], with the initial selection (gate) defined by excluding the debris region. Autofluorescence negative controls were performed using unlabeled cells to serve as a reference for determining the phenotype of *C. thous* MSCs, using CD45 and CD14 as negative markers and CD44, CD90, and CD29 as positive markers.

To confirm the multipotent differentiation potential toward mesodermal lineages, previously cultured cells (P3) were induced to differentiate in vitro into chondrogenic, adipogenic, and osteogenic lineages using the StemPro Adipogenesis^®^, StemPro Chondrogenesis^®^, and StemPro Osteogenesis^®^ kits (Invitrogen, São Paulo, SP, Brazil). After differentiation, the cells were fixed with 4% paraformaldehyde (Sigma-Aldrich, St Louis, MO, USA) and subjected to lineage-specific staining: Alcian Blue for chondrogenic differentiation, Oil Red O for adipogenic differentiation, and Alizarin Red for osteogenic differentiation, according to the manufacturer’s instructions.

### Morphological and cell viability assessment following in vitro inflammatory stimulation

AT-MSCs at P3 were assessed for viability using the Trypan Blue exclusion assay prior to plating. Briefly, cells were then plated in duplicate in 24-well plates (Sarstedt, Nümbrecht, Germany) at a density of 1 × 10⁵ cells/well using standard culture medium composed of DMEM supplemented with 10% FBS, 1% penicillin–streptomycin, and 1% amphotericin B. After 24 h to allow cell adhesion, baseline images were acquired using an inverted microscope (Leica DM IRB, Leica Microsystems, Wetzlar, Germany) immediately before stimulation (T0).

After initial imaging (T0), the culture medium was replaced according to the experimental conditions: control group (standard culture medium) and LPS group (standard culture medium supplemented with 10 ng/mL of lipopolysaccharide), using 500 µL of medium per well. After 24 h under the experimental conditions, a second set of images was acquired (T24). The LPS concentration (10 ng/mL) and the 24 h evaluation time point were selected based on previously established protocols in our research group (Kamura et al. 2026) and published studies demonstrating that these conditions induce inflammatory responses without causing significant cytotoxicity to MSCs (El Fajri et al. 2025; Kim et al. 2016; Yin et al. 2017).

Cells were then trypsinized, centrifuged, and resuspended in 3 mL of DMEM. Subsequently, 20 µL of each cell suspension was mixed with 20 µL of Trypan Blue and homogenized. Ten microliters of the mixture were loaded onto a Neubauer chamber for microscopic quantification of viable and non-viable cells, allowing calculation of their respective percentages.

### Gene expression analysis of cytokines and neurotrophic factors

Gene expression analysis was performed by RT-qPCR in both the control and LPS groups to quantify the following cytokines and neurotrophic factors: interleukin-1 beta (IL-1β), interleukin-2 (IL-2), interleukin-6 (IL-6), interleukin-10 (IL-10), prostaglandin E synthase 2 (PTGES2), indoleamine 2,3-dioxygenase (IDO), tumor necrosis factor-alpha (TNF-α), interferon-gamma (IFN-γ), brain-derived neurotrophic factor (BDNF), glial cell line-derived neurotrophic factor (GDNF), hepatocyte growth factor (HGF), and vascular endothelial growth factor A (VEGF-A).

For RNA isolation, AT-MSCs were transferred in duplicate, lysed with 1 mL of Trizol^®^, and collected for subsequent analyses. Total RNA was extracted according to the manufacturer’s instructions. The RNA was eluted in RNase-free water, quantified, and assessed by spectrophotometry using a Thermo Scientific NanoDrop 2000, and the 260/280 nm and 260/230 nm absorbance ratios were evaluated to ensure the quality of the extracted material. cDNA synthesis was performed using the High-Capacity cDNA Reverse Transcription Kit, following the manufacturer’s recommended protocol. Reverse transcription was carried out in a Veriti 96 Well Thermal Cycler under the following conditions: 10 min at 25 °C, 12 min at 37 °C, and 5 min at 85 °C. PCR reactions were performed in duplicate using the previously synthesized cDNA, PowerUp™ SYBR™ Green Master Mix, and RNase-free water. For data normalization, the reference genes hypoxanthine-guanine phosphoribosyltransferase (HPRT), ribosomal protein S5 (RPS5) and S19 (RPS19) were used. Real-time polymerase chain reaction (qPCR) was performed using the QuantStudio™ 12 K Flex Real-Time PCR System under the following amplification conditions: 50 °C for 2 min, followed by 95 °C for 2 min, and 40 cycles of 95 °C for 1 s and 60 °C for 30 s. At the end of the amplification cycles, a dissociation (melting) curve was generated to verify the specificity of the amplified products.

Primer sequences for the assessment of cytokine and neurotrophic factor gene expression were selected based on homologous regions identified in *Canis lupus familiaris* (Table 1) because genomic data for *Cerdocyon thous *are not available. Sequence conservation among canid species supports the applicability of these primers for gene expression analysis in this species. Following amplification, melting-curve analysis was performed for each reaction to assess product specificity. All primer pairs generated single, sharp melting peaks in *Cerdocyon thous* samples, with no evidence of nonspecific amplification or primer-dimer formation. The corresponding melting curves are provided in Supplementary Material 1.

**Table 1:** Sequences of primers utilized in RT-qPCR assays

DNA-targets	5’>3’
IL-1β	(F)TGAAGTGCTGCTGCCAAGAC
	(R)GCAACTGGATGCCCTCATCT
IL-2	(F)CAACTCCTGCCACAATGTACAAA
	(R)TGCGACAAGTACAAGCGTCAGT
IL-6	(F)GACCACTCCTGACCCAACCA
	(R) ATCCTGCGACTGCAAGATAGC
BDNF	(F) GTGTCGAAAGGCCAACTGAAG
	(R)CGTGTAACCCATGGGATTGC
IDO	(F) TGTGGACCCAAGCACGTTTT
	(R) AGTTGCCTTTCCAACCAGACA
GDNF	(F) GGTTTGCTACAGCCAGCAGTT
	(R) CGCACCATGTTCAAAATCCA
PTGES2	(F) GCCTGCAGCTGACCCTGTA
	(R) CACGGACCTTGCTGCAGAA
HGF	(F) ATGGTTCTTGGCGTCATTGTT
	(R) AATGCCAGGACGATTTGGAA
IL-10	(F) CCCAGGATGGCAACTCTTCTC
	(R) CGGGATGGTATTTTGCAGATC
INF-Ƴ	(F)TCTCACCAAGATCCAACCTAAGG
	(R) TGCGGCCTCGAAACAGA
TNF-α	(F) TGGAATCATTGCCCTGTAAGG
	(R) TGATCAAAGGGTCGGTTTGG
RPS19	(F) GGGTCCTCCAAGCCCTAGAG
	(R) CGGCCCCCATCTTGGT
HPRT	(F) CGGCTTGCTCGAGATGTGAT
	(R) GCACACAGAGGGCTATGT
RPS5	(F) GAGGCCTCAGGCTGTCGAT
	(R) AGCCAAATGGCCTGATTCAC

*IL-1β* Interleukin-1 beta, *IL-2* Interleukin-2, *IL-6* Interleukin-6, *IL-10* Interleukin-10, *BDNF* Brain-Derived Neurotrophic Factor, *GDNF* Glial Cell Line-Derived Neurotrophic Factor, *HGF* Hepatocyte Growth Factor, *HPRT* Hypoxanthine Phosphoribosyltransferase (1) *IDO* Indoleamine 2,3-Dioxygenase, *IFN-γ* Interferon gamma, *PTGES2* Prostaglandin E Synthase (2) *RPS5* Ribosomal Protein S5, *RPS19* Ribosomal Protein S19, *TNF-α* Tumor Necrosis Factor alpha

### Statistical analysis

The experiment was performed using cells isolated from five independent animals (*n* = 5), with each experiment conducted in duplicate. Technical replicates were also used, where appropriate, for each analytical method. All data were assessed for normality using the Shapiro–Wilk test. Data were normally distributed and were analyzed using an unpaired t-test. Statistical analyses and graph generation were performed using GraphPad Prism, version 10.4.1 (GraphPad Software, La Jolla, CA, USA). Differences were considered statistically significant at *p* < 0.05.

## Results

### Isolation and culture of mesenchymal stem cells (MSCs)

The isolation and in vitro expansion of AT-MSCs from *C. thous* specimens were successfully performed. The obtained cell lines demonstrated cellular viability and maintained proliferative potential under controlled culture conditions, confirming the effectiveness of the established protocol.

### Morphological, immunophenotypic, and multipotency characterization

**Fig. 1:**
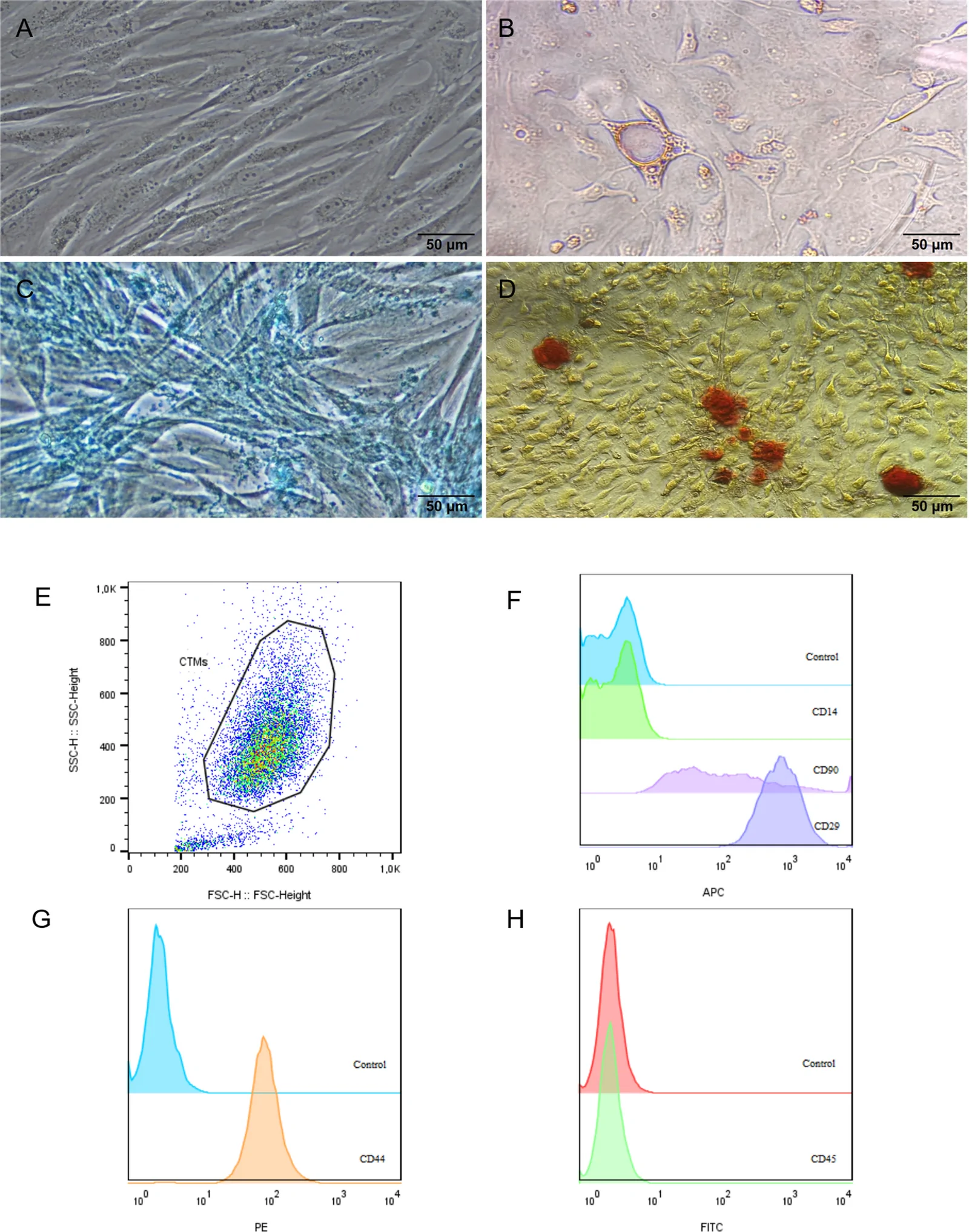
Morphological and immunophenotypic characterization of adipose tissue-derived mesenchymal stem cells (AT-MSCs) from *Cerdocyon thous*. (**A**) fibroblast-like morphology during in vitro expansion (20x magnification); (**B**) adipogenic differentiation stained with Oil Red O (20x magnification); (**C**) chondrogenic differentiation evidenced by Alcian Blue staining (20x magnification); (**D**) osteogenic differentiation stained with Alizarin Red (20x magnification); (**E**) FSC-H versus SSC-H dot plot showing a homogeneous cell population and the gating strategy used to define the mesenchymal stem cell population; (**F**) representative histograms demonstrating high expression of the mesenchymal markers CD90 and CD29 compared with their respective autofluorescent controls (blue curve), and minimal expression of the hematopoietic marker CD14; (**G**) histogram showing high expression of CD44; (**H**) histogram showing low expression of the hematopoietic marker CD45

During the in vitro expansion period, the cells exhibited a typical fibroblast-like morphology, characterized by an elongated shape, abundant cytoplasm, and adherent growth on the plastic surface, maintaining a homogeneous morphological pattern across all evaluated passages (Fig. 1A).

The AT-MSCs from *C. thous* demonstrated multipotent differentiation capacity following in vitro induction, successfully differentiating into adipogenic (Fig. 1B), chondrogenic (Fig. 1C), and osteogenic lineages (Fig. 1D). Cultures subjected to adipogenic induction exhibited progressive accumulation of lipid droplets within the cellular cytoplasm (Fig. 1B). Chondrogenic differentiation resulted in the deposition of glycosaminoglycans in the intra- and extracellular matrix, a characteristic feature of the chondrocytic phenotype (Fig. 1C). Osteogenic differentiation led to the formation of a mineralized extracellular matrix (Fig. 1D). Taken together, these results confirm the mesodermal multipotency of the MSCs evaluated in this study.

Flow cytometry analysis revealed that the cell population exhibited a homogeneous profile, as shown in the FSC-H versus SSC-H dot plot (Fig. 1E), allowing precise gating of the mesenchymal stem cell population. Immunophenotypic evaluation demonstrated expression of the mesenchymal markers CD90 (98.02%) (Fig. 1F), CD29 (99.99%) (Fig. 1F), and CD44 (99.81%) (Fig. 1G). In contrast, absence of expression of the hematopoietic markers CD14 (1.60%) (Fig. 1F) and CD45 (1.81%) (Fig. 1H) was observed, with profiles overlapping their respective controls. These results provide further confirmation of the characteristic immunophenotypic profile of mesenchymal stem cells (MSCs) in the analyzed population. The cross-reactivity of anti-CD14 and anti-CD45 antibodies was confirmed using peripheral blood from *C. thous.*

### Morphological and cell viability assessment following in vitro inflammatory stimulation

**Fig. 2:**
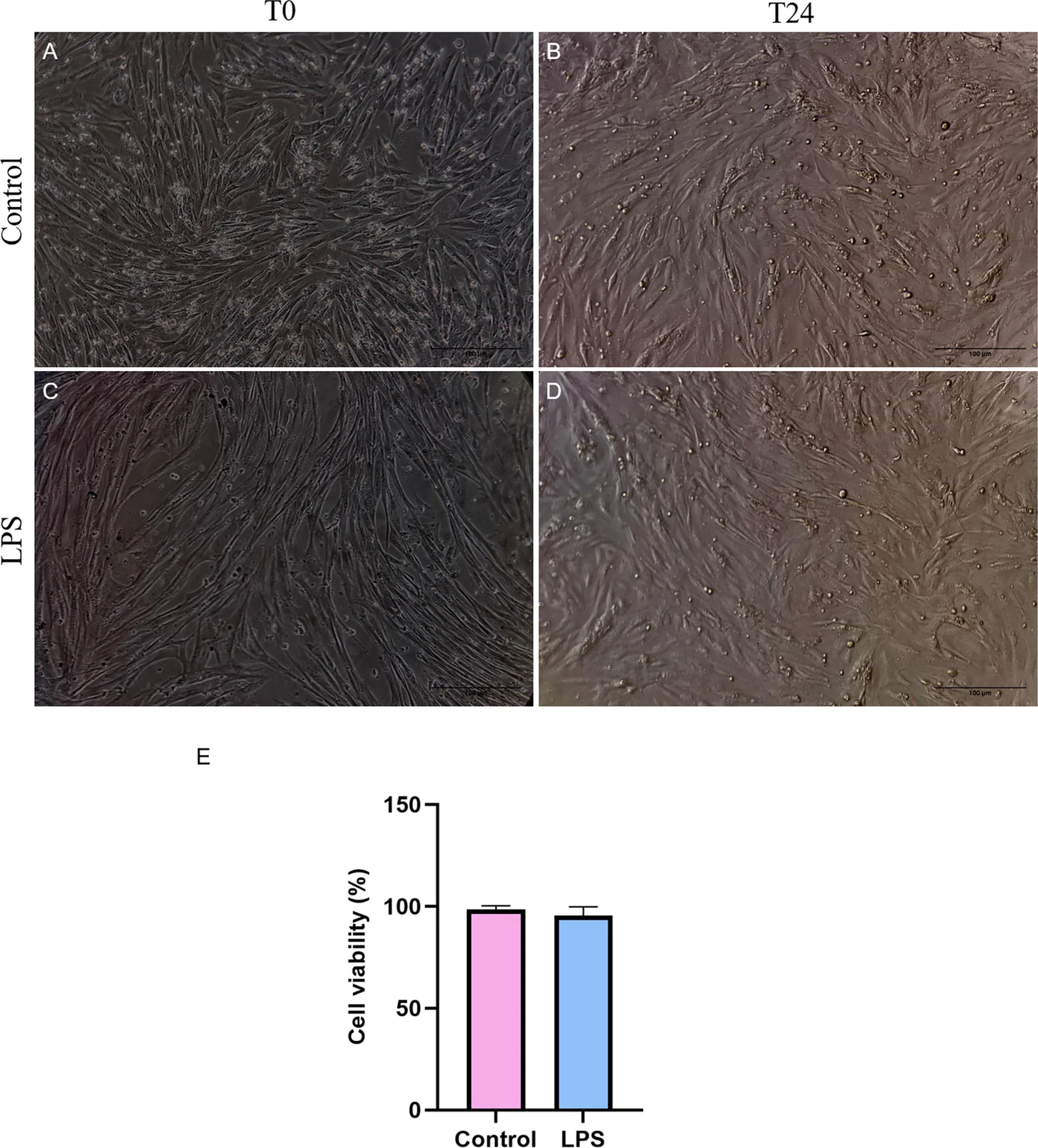
Morphology and cell viability of adipose tissue-derived mesenchymal stem cells (AT-MSCs) from *Cerdocyon thous*. Cell morphology was assessed at two time points: immediately before stimulation (T0) and 24 h post-stimulation (T24). (**A**) control group at T0; (**B**) control group at T24; (**C**) cells stimulated with lipopolysaccharide (LPS) at T0; (**D**) cells stimulated with LPS at T24; (**E**) cell viability 24 h after stimulation. Statistical analysis was performed using an unpaired t-test, showing no significant difference between groups (*p* = 0.1584). Data are presented as mean ± standard deviation from five biological replicates (*n* = 5 animals)

AT-MSCs from *C. thous* showed 99.9% viability prior to plating. After 24 h under control or LPS-stimulated conditions, high viability rates were maintained in both experimental groups, with no statistically significant differences observed (*p* = 0.1584), indicating that the inflammatory stimulus did not exert a cytotoxic effect on the AT-MSCs during the evaluation period (Fig. 2E). No morphological differences were observed between groups; cells maintained typical fibroblast-like appearance and adhered to the plastic surface throughout (Fig. 2A–D).

### Gene expression analysis of cytokines and neurotrophic factors

**Fig. 3:**
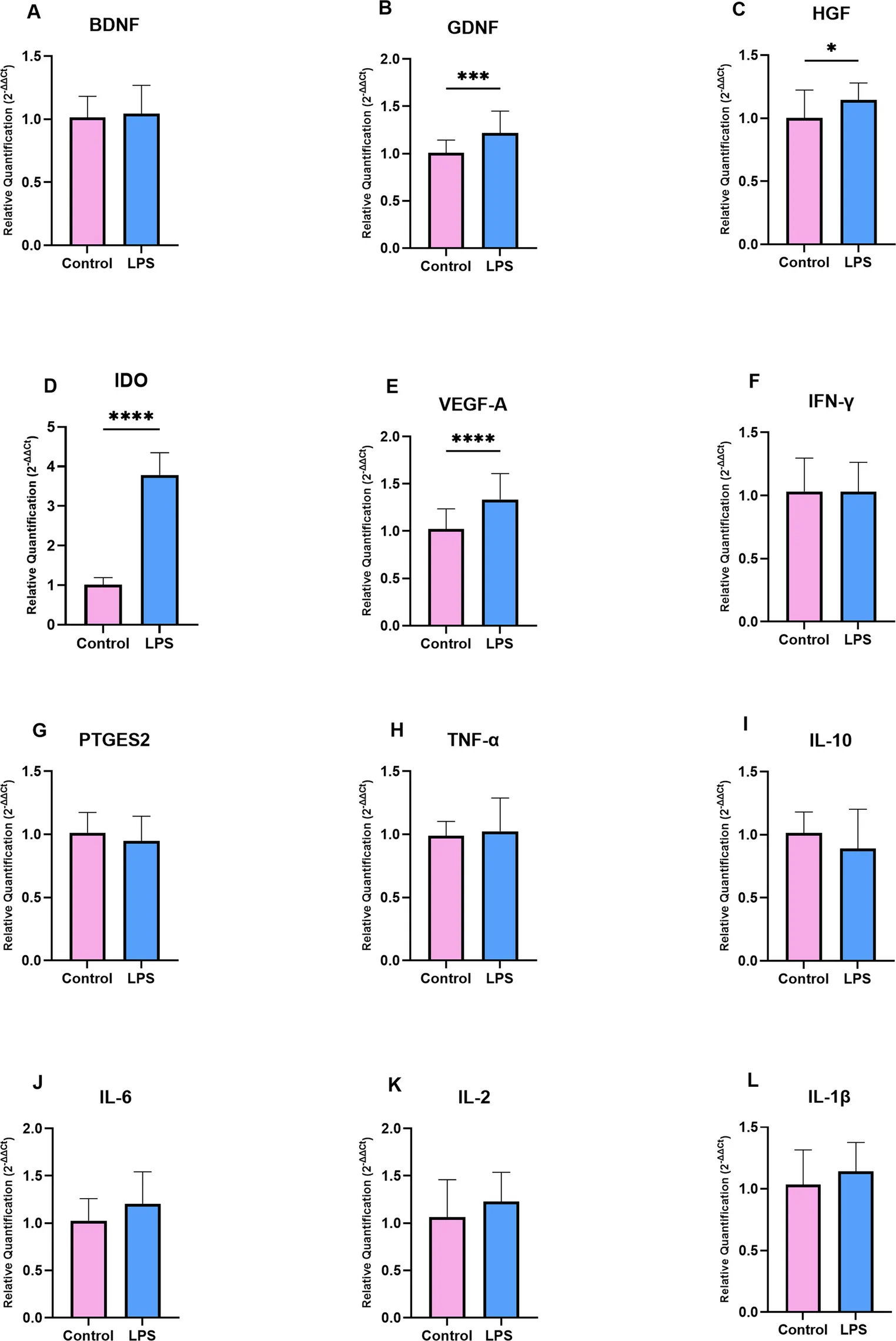
Relative gene expression of inflammatory cytokines and neurotrophic factors in adipose tissue-derived mesenchymal stem cells (AT-MSCs) from *Cerdocyon thous* 24 h after stimulation. Comparisons were made between control and lipopolysaccharide (LPS)-stimulated groups. (**A**) Brain-derived neurotrophic factor (BDNF), (**B**) glial cell line-derived neurotrophic factor (GDNF), (**C**) hepatocyte growth factor (HGF), (**D**) indoleamine 2,3-dioxygenase (IDO), (**E**) vascular endothelial growth factor A (VEGF-A), (**F**) interferon-gamma (IFN-γ), (**G**) prostaglandin E synthase 2 (PTGES2), (**H**) tumor necrosis factor-alpha (TNF-α), (**I**) interleukin-10 (IL-10), (**J**) interleukin-6 (IL-6), (**K**) interleukin-2 (IL-2), and (**L**) interleukin-1 beta (IL-1β). Data are presented as mean ± standard deviation from five independent animals (biological replicates; *n* = 5). Each biological replicate was analyzed in duplicate, and RT-qPCR reactions were performed in technical duplicate. Statistical analysis was performed using an unpaired t-test. Statistical significance was defined as follows: *p < 0.05, ***p < 0.001, and ****p < 0.0001

Melting-curve analysis showed a single, well-defined peak for each of the 12 target genes and three reference genes, supporting the amplification of single predominant products in *Cerdocyon thous* samples. A significant increase in GDNF expression was observed in cells stimulated with LPS (10 ng/mL) compared to the control group (*p* = 0.0004) (Fig. 3B). Similarly, HGF expression was significantly elevated after inflammatory stimulation (*p* = 0.0103) (Fig. 3C). No differences in BDNF expression were observed between the analyzed groups.

Analysis of immunological mediators revealed a marked and statistically significant increase in IDO gene expression in the LPS-stimulated group compared to the control group (*p* < 0.0001/Fig. 3D). Additionally, expression of the angiogenic factor VEGF-A was significantly elevated in LPS-stimulated cells relative to the control (*p* < 0.0001/Fig. 3E). In contrast, no statistically significant differences were observed in the expression of IL-10, IL-6, IL-2, IL-1β, IFN-γ, TNF, or PTGES2 between the evaluated groups (Fig. 3F-L).

## Discussion

To our knowledge, this study provides the first description and characterization of MSCs derived from *Cerdocyon thous*, expanding the current knowledge of stem cell biology in wild canids. AT-MSCs from *C. thous* exhibited morphological, immunophenotypic, and functional characteristics consistent with the minimal criteria established by the International Society for Cellular Therapy for defining of MSCs (Dominici et al. 2006). Cell adherence to plastic, fibroblast-like morphology, expression of mesenchymal markers, lack of hematopoietic marker expression, and capacity for adipogenic, chondrogenic, and osteogenic differentiation confirm that the analyzed cell population represents a multipotent mesenchymal lineage (Dominici et al. 2006). This aspect is essential to ensure that the responses observed following inflammatory stimulation can be attributed to the intrinsic properties of MSCs rather than to the presence of heterogeneous cell populations.

The response of AT-MSCs to LPS revealed a functional activation pattern more closely associated with immunomodulation than with classical inflammation (Cagliani et al. 2017). In many immune cells, LPS acts as a potent inducer of pro-inflammatory responses through Toll-like receptors (TLRs), particularly TLR4 (Luo et al. 2025). However, in MSCs, exposure to inflammatory signals can trigger the expression of regulatory mediators that suppress excessive immune activation rather than amplifying it directly. Stimulation with LPS at 10 ng/mL resulted in significant upregulation of IDO and VEGF-A, whereas pro- and anti-inflammatory cytokines, including IL-10, IL-6, IL-2, IL-1β, IFN-γ, TNF-α, and PTGES2, remained unchanged. This pattern may reflect the relatively mild inflammatory stimulus provided by this concentration, as higher LPS doses (e.g., 100 ng/mL) have been reported to induce more robust cytokine responses in MSCs (Noh et al. 2016). Collectively, these findings highlight the adaptive immunomodulatory properties of MSCs, in which pro-inflammatory stimuli promote the expression of immunoregulatory molecules that act within the microenvironment to balance immune responses (Cagliani et al. 2017; Hoseinzadeh et al. 2025).

The maintenance of AT-MSC viability following inflammatory stimulation indicates that these cells can tolerate pro-inflammatory environments without compromising their cellular integrity. This behavior aligns with the concept of “inflammatory licensing” of MSCs (Krampera 2011), according to which exposure to inflammatory mediators does not exert cytotoxic effects but acts as a functional cue that modulates the secretory and immunological profile of these cells (Krampera 2011; Waterman et al. 2010). MSCs are relevant for applications in neurological disorders (Issa et al. 2025), as inflammatory licensing has been associated with increased secretion of neurotrophic factors such as BDNF, NGF, GDNF, and IGF-1, which play critical roles in neuroprotection, synaptic plasticity, and axonal regeneration (Crigler et al. 2006; Uccelli et al. 2008; Shi et al. 2018).

Among the mediators evaluated, the robust induction of IDO expression was a central component of the regulatory response of MSCs. IDO is a key enzyme involved in tryptophan catabolism via the kynurenine pathway, which can result in local tryptophan depletion and the production of metabolites that inhibit T lymphocyte proliferation and activation, thereby promoting immune tolerance and limiting the propagation of excessive inflammatory responses (Gao et al. 2016; Jiang and Xu 2020). Cagliani et al. (2017) demonstrated that, although IDO is not typically constitutively expressed in MSCs, its expression can be strongly induced by inflammatory signals, particularly IFN-γ, leading to relevant immunosuppressive effects in both experimental and clinical models. However, the contribution of IDO to MSC-mediated immunosuppression appears to be species-dependent. In domestic canine MSCs, functional studies have shown that inhibition of the IDO pathway does not significantly reverse T-cell suppression, suggesting that, unlike human MSCs, IDO is not the predominant immunoregulatory mechanism. Instead, canine MSCs appear to rely mainly on TGF-β, adenosine and cyclooxygenase/PGE₂ pathways to suppress T-cell responses (Chow et al. 2017). Therefore, the increased IDO expression observed following LPS stimulation in the present study should be interpreted as evidence of inflammatory priming and activation of MSCs rather than as evidence that IDO is the principal mediator of their immunosuppressive activity. Although functional studies in domestic canines suggest that IDO is not the predominant mechanism of MSC-mediated immunosuppression, whether this also applies to MSCs derived from wild canids remains unknown.

In the context of neural regeneration and tissue support, a significant increase in neurotrophic factors such as GDNF and HGF was observed, both recognized for their ability to promote neuronal survival and reparative properties. GDNF, which is essential for maintaining the integrity and development of dopaminergic neurons, is synthesized by microglial cells (Aldhshan et al. 2022) and enhances motor neuron survival, supports axonal regeneration and improves muscle reinnervation (Lavorato, 2021; Aldhshan et al. 2022). Similarly, HGF exerts mitogenic, anti-apoptotic, and tissue repair-promoting effects across various cellular models, including modulation of immune responses and expansion of regulatory cell populations, as demonstrated in human mesenchymal stem cells (Yen et al. 2013).

Furthermore, the increase in VEGF-A suggests an angiogenic component in the MSC response to LPS. VEGF-A is a key mediator of new blood vessel formation, contributing to improved tissue perfusion, removal of inflammatory metabolites, and support of a regenerative microenvironment. MSCs can secrete a range of pro-angiogenic and immunomodulatory factors in response to inflammatory or hypoxic stimuli, supporting the hypothesis that their therapeutic properties are largely mediated by their paracrine secretome rather than by direct differentiation (Madrigal et al. 2014).

Taken together, these findings suggest that AT-MSCs from *C. thous* not only act as modulators of the immune environment under pro-inflammatory stimulation but also express factors capable of shaping the microenvironment for tissue repair and neuroprotection, including molecules with proangiogenic, neurotrophic, and immunoregulatory activities. This combination aligns with the emerging paradigm that the MSC secretome represents a major mediator of their therapeutic potential, influencing neighboring cells and remodelling the local microenvironment (Gnecchi et al. 2008; Colbath et al. 2024).

From a translational perspective, the results of this study are further relevant because they were obtained from a wild species model. This is particularly notable given that studies involving MSCs in non-conventional species remain limited and are distributed across diverse vertebrate lineages. Previous investigations have described MSCs or MSC-like cells in amphibians (*Xenopus laevis*) (Otsuka-Yamaguchi, 2021), reptiles (*Trachemys scripta*) (Andreoli et al. 2025), and, more broadly, in placental mammals, including rodents (*Ctenomys minutus*) (Pereira et al. 2018), bats (*Artibeus planirostris*) (Carvalho et al. 2021), wild artiodactyls (*Tayassu tajacu*) (Argôlo Neto et al. 2016), aquatic mammals (*Tursiops truncatus*) (Griffeth et al. 2014), and large herbivores (*Hippopotamus amphibius*) (Wei et al. 2022) and omnivorous mammals (*Ailurus fulgens*) (Wang et al. 2022).

Within mammals, additional studies have included non-human primates (Kang et al. 2025) such as *Macaca fascicularis* (Rinendyaputri et al. 2026), *Macaca mulatta* (Poret et al. 2021) and *Callithrix jacchus* (Kanda et al. 2013), as well as representatives of the order Carnivora, including *Leopardus guigna* (Echeverry et al. 2019), *Puma concolor* (Echeverry et al. 2020), and *Mustela lutreola* (Calle and Ramírez 2022). Nevertheless, to our knowledge, no studies have systematically described the morphological behavior, functional properties, or immunomodulatory and neuroregenerative profiles of MSCs derived from *C*. *thous*. Demonstrating that these cells exhibit properties comparable to those reported in other species significantly expands the knowledge base necessary for future applications in conservation veterinary medicine and for the study of naturally occurring diseases in wild animal models.

Despite these promising findings, several important limitations must be considered. This study assessed only the gene expression of selected mediators without directly measuring protein levels or the ultimate functional effects of these factors. In addition, the inflammatory response was evaluated at a single time point (24 h), which may not fully capture the temporal dynamics of MSC responses to inflammatory stimulation. Future studies incorporating multiple evaluation time points, larger sample sizes, together with proteomic analyses, co-culture assays, injury models, and in vivo functional evaluations are essential to determine whether the observed expression patterns translate into consistent and biologically relevant effects.

## Conclusion

Based on the results obtained, AT-MSCs from *C. thous* exhibit morphological and immunophenotypic characteristics consistent with MSCs, including fibroblast-like morphology, expression of mesenchymal markers, absence of hematopoietic markers, adipogenic, chondrogenic, and osteogenic differentiation capacity, and maintenance of viability following inflammatory stimulation. Furthermore, LPS exposure modulated the expression of genes associated with immunoregulatory and regenerative mechanisms, as evidenced by the significant upregulation of IDO and neurotrophic and angiogenic factors, such as GDNF, HGF, and VEGF-A. These findings provide the first characterization of AT-MSCs from *C. thous* and demonstrate their ability to respond to inflammatory stimulation in vitro by modulating the expression of genes associated with immunoregulatoy and neuroregenerative pathways. These results establish functional properties and potential applications of MSCs in conservation veterinary medicine, particularly for inflammatory and neurological disorders.

## Supplementary Information

Below is the link to the electronic supplementary material.

Supplementary Material 1 (DOCX 785 KB)

## Data Availability

The datasets used and/or analysed during the current study are available from the corresponding author on reasonable request.
