# Supplementary material for: Mesenchymal stem cells from Cerdocyon thous: in vitro characterization and therapeutic potential
Source: Vet Res Commun. 2026 Sep 26;50(6):598. doi: 10.1007/s11259-026-11542-4 (PMC13615927; doi:10.1007/s11259-026-11542-4)
Supplement: Supplementary file 1 [file 11259_2026_11542_MOESM1_ESM.docx]

Supplementary Material 1: Following amplification, melting-curve analysis was performed for each reaction to assess product specificity. All primer pairs generated single, sharp melting peaks in Cerdocyon thous samples, with no evidence of nonspecific amplification or primer-dimer formation.
